# Calcifying nanoparticles: one face of distinct entities?

**DOI:** 10.3389/fmicb.2014.00214

**Published:** 2014-05-20

**Authors:** Anton G. Kutikhin, Arseniy E. Yuzhalin, Vadim V. Borisov, Elena A. Velikanova, Alexey V. Frolov, Vera M. Sakharova, Elena B. Brusina, Alexey S. Golovkin

**Affiliations:** ^1^Division of Experimental and Clinical Cardiology, Research Institute for Complex Issues of Cardiovascular Diseases under the Siberian Branch of the Russian Academy of Medical SciencesKemerovo, Russia; ^2^Department of Epidemiology, Kemerovo State Medical AcademyKemerovo, Russia; ^3^Central Research Laboratory, Kemerovo State Medical AcademyKemerovo, Russia; ^4^Department of Oncology, Cancer Research UK and Medical Research Council Oxford Institute for Radiation Oncology, University of OxfordOxford, UK

**Keywords:** calcifying nanoparticles, nanobacteria, nanobacteria-like particles, calcification, inflammation, atherosclerosis, diseases, hydroxyapatite

Calcifying nanoparticles (CNPs, nanobacteria, nanobacteria-like particles) were discovered as cell culture contaminants by Kajander et al. more than 25 years ago, and the first results of their work were published some years later (Kajander et al., 1997; Kajander and Ciftçioglu, 1998). The nature of CNPs has been obscured so far. Possibly representing a new proposed class of living organisms or inorganic nanostructures, CNPs exist as coccoid, coccobacillar, or bacillary particles of 80–500 nm in diameter, consisting of a central cavity surrounded by the hydroxyapatite shell, and possessing an ability to grow and divide in culture medium forming biofilms. The phenomenon of CNPs raised intensive discussions amongst the scientific community; there are active debates on their nature and potential role in clinical medicine. According to a number of fundamental and clinical studies, CNPs are suspected to cause ectopic calcification-related diseases such as atherosclerosis, heart valve calcification, placental calcification, nephrolithiasis, cholecystolithiasis, type III chronic prostatitis/chronic pelvic pain syndrome, and testicular microlithiasis. Electron microscopy is considered to be a gold standard for the visualization of CNPs; researchers usually observe the colonies of CNPs after the culturing in DMEM or RPMI-1640 for 4–8 weeks. Other means used by multiple groups for the detection of CNPs are various serological methods, including ELISA, immunohistochemistry, immunofluorescence reaction, immunoblotting, and Ouchterlony immunodiffusion. Although PCR has been previously used to identify possible genome of CNPs, there are doubts on credibility of this method since primers could have been designed based not on putative genome sequences of CNPs but on the genome sequences of contaminating bacteria. The general features of CNPs are indicated in Table 1.

**Table 1:** **Properties of calcifying nanoparticles (CNPs)**.

	**Properties of calcifying nanoparticles**
Morphological properties	80–500 nm in diameter (can pass through 100-nm filters)
	Usually have coccoid, coccobacillar, or bacillar form, often remind mineralized «igloo»
	Have hydroxyapatite shell, cellular-membranous structure, and central cavity
	Can form microscopic colonies (>1 mm in diameter) under the low concentration of nutrients in the environment
	Divide by binary fission, fragmentation, and gemmation
	Can form thermoresistant biofilms
Tinctorial properties	Gram-negative, can be stained by DNA-specific dyes (Hoechst 33258 at a concentration of 5 μg/ml during 50 min, propidium iodide, PicoGreen—they are specific after the filtration through 0.1–0.22 μm pores, staining after a demineralization is optimal), can be revealed by von Kossa staining, staining by 2% uranyl acetate (possibly with lead citrate) can detect specific mucus on the hydroxyapatite shell, in the mineralized state can be stained by alizarin red S, can be stained by phosphotungstic acid
Resistance and sensitivity	Resistant to 90°C heating during 1 h
	Resistant to γ-irradiation up to 30 kGray
	Resistant to 5% NaCl solution
	In the mineralized state are resistant to lyzozyme, proteinase K, certain other proteinases, lipases, amylases, alkali, ultrasound, X-ray, detergents, and solvents
	Temperature under 37°C suppresses replication and prevents biofilms formation
	Resistant to wide spectrum of antimicrobial therapeutics: aminoglycosides (in pharmacological concentrations), chloramphenicol, lincosamides, cephalosporins, macrolides, fluoroquinolones, glycopeptides (in pharmacological concentrations), polymyxins, antituberculous agents, aminocyclitol, spectinomycin
	Sensitive to tetracyclines, ampicillin, trimethoprim, trimethoprim-sulfamethoxazole, nitrofurantoin, 5-fluorouracil, cytosine arabinoside, antimycin A, sodium azide, potassium cyanide, bisphosphonates (etidronate, clodronate), 6-aminocaproic acid, *in vitro* growth can be inhibited by EDTA, EGTA, and citrate
Culture properties	Doubling time is 3 days, in media without serum—6 days
	Passage can be performed in DMEM (Dulbecco's modified Eagle's medium) or RPMI-1640 independently of the presence of serum
	Optimal atmosphere for growth should contain 5% of CO_2_ and 95% of air
	Sensitive to β-mercaptoethanol, which stimulates growth of anaerobes, but cannot be cultivated under the strictly anaerobic conditions
	Calcify when serum concentration in the culture media is lower than 5%
	Cytotoxic for fibroblasts and lymphocytes
Biochemical properties	Metabolism is 10,000 times slower than in *E. coli*
	Incorporate uridine (into the expected nucleic acid), methionine, and aspartic acid (into the expected system of protein biosynthesis)
	Calcify under the physiological pH (7.4)
	Urease negative
Detection methods	Bacterioscopic (DNA-specific dyes Hoechst 33258, propidium iodide, PicoGreen, staining after a demineralization is optimal) using scanning and transmission electron microscopy, von Kossa staining which is specific for calcium compounds, staining by 2% uranyl acetate (possibly with lead citrate) to detect specific mucus on the hydroxyapatite shell, staining by alizarin red S in the mineralized state, staining by phosphotungstic acid, after the long-term cultivation light microscopy with von Kossa staining is possible to be used for detection
	Bacteriological (cultivation in DMEM or RPMI-1640 without serum under 37°C during 4–6 weeks after the filtration through 0.1–0.22 μm pores), replication can be assessed by spectrophotometry (650 nm wavelength)
	Serological CNP antigens and anti-CNP monoclonal antibodies 8/0 (to porin), 5/2 (to peptidoglycan) and 8D10 (to porin) of NanoBiotech Pharma, Tampa, FL, USA—ELISA, immunohistochemistry, immunofluorescence reaction, immunoblotting, Ouchterlony immunodiffusion
	Genomic (PCR), but there is doubt that existing primers are obtained on the basis of CNP nucleic acids, and not on the basis of nucleic acids of contaminating bacteria
	Proteomic (sodium dodecyl sulfate polyacrylamide gel electrophoresis with further identification of protein bands by mass spectrometry)
Methods of treatment of CNP-associated diseases (atherosclerosis, heart valve calcification, placental calcification, nephrolithiasis, cholecystolithiasis, type III chronic prostatitis/chronic pelvic pain syndrome, and testicular microlithiasis)	comET-therapy (tetracycline, EDTA, and mix of nutrients)

*Abbreviations: CNPs, calcifying nanoparticles; EDTA, ethylenediaminetetraacetic acid; EGTA, ethyleneglycoltetraacetic acid; DMEM, Dulbecco's modified Eagle's medium; RPMI-1640, Roswell Park Memorial Institute 1640 medium; ELISA, enzyme-linked immunosorbent assay; PCR, polymerase chain reaction. Cited from Chang et al. (1996)*.

Since the beginning of 2012, when our group published a comprehensive review on the role of CNPs in biology and medicine (Kutikhin et al., 2012), a number of newer findings have been reported. Currently, it is possible to divide all researchers studying CNPs into three groups: (1) those that presume that CNPs are living organisms (nanobacteria), (2) those that consider them as culprits of pathological calcification, and (3) those that completely reject their pathological role and assume them as the result of physiological calcification.

A group from China investigated the role of CNPs in placental calcification. The authors successfully isolated and cultured CNPs from the majority of calcified placental samples but not from normal placental tissues, concluding that CNPs are associated with this pathology (Guo et al., 2012; Lu et al., 2012a,b). Further, the authors also detected «nucleic acid-like materials» within individual CNPs in consistency with previous results by Agababov et al. (2007). Despite the fact that sequencing of putative nanobacterial 16s rRNA was successful, the obtained sequences were 99% similar to *Agrobacterium tumefaciens* and *Rheinheimera* spp., which suggests sample contamination (Guo et al., 2012; Lu et al., 2012a,b). In addition, the researchers speculated that genome of CNPs isolated from calcified placental samples may differ from the putative genome of CNPs isolated from human blood and other sources (Guo et al., 2012); nevertheless, it is arguable that this assumption can be proved by *in silico* analysis whether CNPs have nucleic acids or not.

The same research group recently carried out a first investigation of the impact of nanohydroxyapatites (nHAPs) and CNPs on growth and viability of cancer cells (Zhang et al., 2014). Both nHAPs and CNPs had considerable cytotoxic effects on MDA-MB-231 breast cancer cell line, including altered size and morphology, formation of large cytoplasmic vacuoles, inhibition of proliferation, and induction of apoptosis (Zhang et al., 2014). Importantly, CNPs caused more pronounced cytotoxic effects as compared to nHAPs, as they induced both early and late apoptosis and necrosis, whereas nHAPs triggered early apoptosis only (Zhang et al., 2014). After the internalization of CNPs by cancer cells, cell shrinkage, chromatin condensation, nuclear fragmentation, nuclear dissolution, and the formation of apoptotic bodies were detected, although nHAPs did not exhibit such an effect (Zhang et al., 2014). No cytotoxic effects were observed in the untreated control group (Zhang et al., 2014).

Another investigation on the role of CNPs in calcification-related diseases was performed by a group from Spain concerning aortic valve calcification (Barba et al., 2012). The authors successfully cultured and isolated CNPs from calcified aortic valves but not from uncalcified control valves, showing a feasible pathological role of CNPs; however, no metabolic activity was observed in the samples, and authors failed to detect CNPs' presupposed nucleic acids by real-time PCR (Barba et al., 2012).

Other researchers did not attempt to extract CNPs from clinical specimens; instead, they investigated the inorganic properties of CNPs and conditions of their formation. Wu et al. (2013a) demonstrated that various charged elements and ions may form mineralo-organic nanoparticles (so-called bions) with bacteria-like morphology and similar properties in biological fluids. Upon formation, bions precipitated with phosphate, accumulated carbonate apatite, incorporated additional elements and thus reflected the ionic milieu of the biological fluid in which they formed (Wu et al., 2013a). Bions were able to increase in size and number and to be sub-cultured in fresh culture medium (Wu et al., 2013a). So, many morphological and cultural features of bions are similar to ones typical for CNPs. The authors suggested that bions may represent a part of a physiological cycle that regulates the function, transport, and disposal of mineral ions in the body, and the accumulation of bions may cause pathological processes in the human body under the conditions of altered calcium homeostasis and disturbed clearance mechanisms (Wu et al., 2013a). So, a proposed hypothesis was that bions may form under both physiological and pathological conditions (Wu et al., 2013a). In addition, the same research group suggested that membrane vesicles secreted by various cells may contain a number of serum proteins and can induce hydroxyapatite precipitation during incubation in cell culture medium forming nanostructures which morphologically resemble CNPs (Wu et al., 2013b). Treatment of these membrane vesicles with anti-phosphatidylserine antibodies resulted in decrease of their mineral seeding activity suggesting that phosphatidylserine may provide nucleating sites for calcium phosphate deposition on the vesicles (Wu et al., 2013b).

Using dynamic light scattering, Peng et al. (2013) showed that serum and ion concentrations within the physiological range may form nanoparticles below 100 nm in diameter which can be phagocytosed by macrophages in a size-independent manner. However, only large nanoparticles or their aggregates were able to induce the production of mitochondrial reactive oxygen species, caspase-1 activation, and secretion of interleukin-1β and therefore cause inflammation (Peng et al., 2013). In addition, the authors found that the set of particle-bound proteins does not depend on particle size and curvature (Peng et al., 2013). According to the work of Baum et al. (2012), aggregates of hemoglobin and various salts from physiological environment can also produce morphological structures resembling CNPs. Finally, Kumon et al. investigated one of the original CNP isolates from urinary stones (P-17) (Kumon et al., 2014). The authors developed anti-P-17 IgM monoclonal antibodies, CL-15, which were specific for oxidized lipids, and combined immunoelectron microscopy with ultrastructural and elemental analysis (Kumon et al., 2014). They suggested that lamellar structures consisted of acidic/oxidized lipids provided structural scaffolds for carbonate apatite and that lipid peroxidation induced by γ-irradiation of fetal bovine serum (FBS) was a major cause of CNP propagation (Kumon et al., 2014). Moreover, it was proposed that oxidized lipids may be a common platform for ectopic calcification in atherosclerosis-prone (ApoE^−/−^) mice, thus CNPs were suggested to be by-products rather than etiological agents of chronic inflammation (Kumon et al., 2014). However, it was also noted that propagation of CNPs largely depended on the amount of oxidized lipids available and therefore could play a role in disease progression (Kumon et al., 2014).

However, it should be clearly stated that distinct processes may lead to the formation of nanostructures which are morphologically similar. Bions, hemoglobin-salt aggregates and oxidized lipids with acidified functional groups may lead to the occurrence of nanomorphological phenomenon called CNPs; however, there is no reason to discount many studies where CNPs were significantly associated with ectopic calcification-related diseases. In patients with these diseases, CNPs were detected significantly more frequently by electron microscopy and cultural features in comparison with control samples. Therefore, despite the fact that the emergence of CNPs can be due to physiological processes such as hemoglobin-salt aggregation, they can also form in greater numbers under pathological conditions such as alteration of metabolic and mineral ion homeostasis or lipid peroxidation regardless of their nature, in this case being the culprits of the ectopic calcification-related diseases. In addition, as demonstrated by Peng et al. (2013), large CNPs or their aggregates may cause chronic inflammation which plays a major role in the development of all these diseases. They should not be by-products of inflammation since: (a) injection of CNPs caused artery calcification (Schwartz et al., 2008), nephrolithiasis (Hu et al., 2010), cholecystolithiasis (Wang et al., 2006), and type III chronic prostatitis (Shen et al., 2010) in animal models; (b) treatment of CNPs by comET-therapy (tetracycline HCl, EDTA, and mixture of nutrients) led to significant decline of CNP detection rate, decreased calcification, and substantial therapeutic improvement in patients with coronary artery disease (CAD) and type III chronic prostatitis/chronic pelvic pain syndrome (Maniscalco and Taylor, 2004; Shoskes et al., 2005; Zhou et al., 2008). So, the phenomenon of CNPs definitely should be taken into account when we talk about the etiology and pathogenesis of ectopic calcification-related diseases.

Regarding the hypothesis of CNPs as of the smallest self-replicating life form on Earth (nanobacteria), the absence of a fairly accurately sequenced genome aborts all discussions about their putative living nature. Nevertheless, the diverse nature of CNPs (bions, hemoglobin-salt aggregates, products of lipid peroxidation, calcified membrane vesicles, other inorganic entities) still leaves a place for speculations that certain nanoscale organisms may also be one of the entities of CNPs. However, this suggestion should be interpreted with caution.

From our point of view, immunological and cytotoxic properties of CNPs are still underinvestigated in the light of their potential pathogenic role. The investigations of Zhang et al. (2014) and Peng et al. (2013) are good examples of this kind; however, new studies are clearly needed, particularly due to increasing role of nanomedicine in clinical practice (nanovesicles for drug delivery, nanobiosensors for disease diagnosis, therapeutic nanoparticles which possibly may act as nucleation agents, etc.). Possibly, B cells may produce antibodies to CNPs, at least to large CNPs and their aggregates, and it is necessary to clarify this issue due to its feasible importance for understanding of the immune response against CNPs and for their serological detection. Moreover, anti-CNP antibodies may be of distinct structure due to feasibly different entities of CNPs, their different size and different proteins coating them. In addition, the significance of CNPs as etiological agents of ectopic calcification-related diseases may be also tested in animal models and clinical trials using anti-CNP treatment. Notwithstanding, current standards of CAD and peripheral artery disease (PAD) therapy do not include drugs specifically directed against the calcification. Unfortunately, comET-therapy which demonstrated certain clinical efficacy in treatment of CAD and type III chronic prostatitis/chronic pelvic pain syndrome should not be used for a prolonged treatment due to the increasing hazard of antibiotic resistance. Possibly, larger clinical trials of comET-therapy analogues that do not contain antibiotics but include other calcium-chelating agents can be worthwhile.

To conclude, these two years have revealed some new facts about CNPs:

- CNPs can be formed by distinct entities such as bions, hemoglobin-salt aggregates, products of lipid peroxidation, calcified membrane vesicles, other inorganic entities, and possibly by living organisms; all these entities may possess similar looking nanomorphological structure;
- CNPs can be formed both under the both physiological and pathological conditions depending on their entity; impairment of metabolic and mineral ion homeostasis and lipid peroxidation may cause increased propagation of CNPs in the human body that may be a direct cause of ectopic calcification-related diseases;
- Large CNPs or their aggregates may cause chronic inflammation by the enhanced production of mitochondrial reactive oxygen species, cytokines, and by activation of pro-apoptotic enzymes;
- CNPs and nHAPs possess a number of cytotoxic effects on cancer cell lines such as alteration of cell size and morphology, inhibition of proliferation, and induction of apoptosis;
- Placental calcification may be definitely added to the list of CNP-caused pathologies.

What is clear is that at the present time there is no universal theory that can entirely characterize CNPs, their characteristics, and biological/medical significance. We should not disclaim the fact that CNPs may form under the physiological conditions; however, there is irrefutable evidence that an appearance of CNPs in the living organism may cause ectopic calcification-related diseases, and CNPs definitely should not be considered as just physiological phenomenon. In addition, the fact that CNPs are more likely to be inorganic structures than life forms definitely should not cause underestimation of their potential pathogenic role. No doubt, further investigations will shed light on the nature of CNPs, their biological properties, and their role in clinical medicine.

## Conflict of interest statement

The authors declare that the research was conducted in the absence of any commercial or financial relationships that could be construed as a potential conflict of interest.
